# Impact of train/test sample regimen on performance estimate stability of machine learning in cardiovascular imaging

**DOI:** 10.1038/s41598-021-93651-5

**Published:** 2021-07-14

**Authors:** Vikash Singh, Michael Pencina, Andrew J. Einstein, Joanna X. Liang, Daniel S. Berman, Piotr Slomka

**Affiliations:** 1 University of California at Los Angeles, Los Angeles, CA USA; 2 Division of Artificial Intelligence in Medicine, Departments of Medicine and Cardiology, Cedars Sinai Medical Center, Beverly Boulevard, Ste. A047N, Los Angeles, CA 8700 USA; 3 Duke Clinical Research Institute, Durham, NC USA; 4 Division of Cardiology, Department of Medicine, Columbia University Irving Medical Center, New York, NY USA; 5 Department of Radiology and Herbert Irving Comprehensive Cancer Center, Columbia University Irving Medical Center, New York, NY USA

**Keywords:** Statistics, Machine learning, Computational biology and bioinformatics, Cardiology

## Abstract

As machine learning research in the field of cardiovascular imaging continues to grow, obtaining reliable model performance estimates is critical to develop reliable baselines and compare different algorithms. While the machine learning community has generally accepted methods such as k-fold stratified cross-validation (CV) to be more rigorous than single split validation, the standard research practice in medical fields is the use of single split validation techniques. This is especially concerning given the relatively small sample sizes of datasets used for cardiovascular imaging. We aim to examine how train-test split variation impacts the stability of machine learning (ML) model performance estimates in several validation techniques on two real-world cardiovascular imaging datasets: stratified split-sample validation (70/30 and 50/50 train-test splits), tenfold stratified CV, 10 × repeated tenfold stratified CV, bootstrapping (500 × repeated), and leave one out (LOO) validation. We demonstrate that split validation methods lead to the highest range in AUC and statistically significant differences in ROC curves, unlike the other aforementioned approaches. When building predictive models on relatively small data sets as is often the case in medical imaging, split-sample validation techniques can produce instability in performance estimates with variations in range over 0.15 in the AUC values, and thus any of the alternate validation methods are recommended.

## Introduction

Machine learning (ML) is a field at the intersection of computer science and statistics which focuses on the identification of patterns in potentially large high dimensional datasets, which can be used to predict various outcomes on unseen data. While machine learning has a variety of useful applications, it is particularly effective in medicine in where it can be used to provide predictions about both the diagnosis and prognosis of various diseases from clinical data sets as well as to guide optimal patient treatment^1^. These predictions are based on analyses of both nominal and numerical risk factors/features (which can be extracted from medical imaging results if applicable), exported as probabilistic outputs, which serve as the basis for classification. The measured performance of various models and the confidence with which they can be applied are influenced by the model validation technique, which assesses how the results of statistical analysis will generalize to an independent data set^2^.

The choice of validation method is pivotal when analyzing the performance of predictive models, especially in medicine when comparing various algorithms using relatively small sample sizes^3^. Variation of the random seed (the separation of the data into training and testing sets), influences the model produced. Consequently, this influences the AUC (area under receiver operator characteristic (ROC) curve) used to measure model performance^4^. While some variation in AUC is to be expected that can be attributed to random seed, to ensure reliable comparisons this variation should not be statistically significant. We aimed to examine how train-test split variation impacts the measured AUC in several validation techniques, namely: split-sample validation (70/30 and 50/50 train-test splits), bootstrap validation, tenfold stratified cross-validation (CV) and 10 × repeated tenfold stratified CV.

## Methods

### Software

These experiments were performed utilizing algorithms from Python’s Sci-Kit Learn library^5^. For each algorithm (logistic regression, gaussian naïve bayes, linear discriminant analysis, and random forest), bootstrap validation, 50/50 stratified split validation, 70/30 stratified split validation, tenfold stratified CV, and 10 × repeated tenfold stratified CV were implemented across 100 different seeds (splits of the data). The splits were consistent across the various algorithms for the sake of consistent evaluation. Bootstrap validation was implemented to sample all instances with replacement as the training set until the number of samples matched the total size of the dataset. A validation set was created from every instance not used in the training set, making the size of the validation set variable on how much replacement occurred in the training set selection. This process was done 500 times per iteration and the average of the AUCs was computed for all the bootstrap samples. A fixed random state was used to control for the random effects associated with the bootstrapping of samples when building trees and sampling of features for splits in random forest models.

### Datasets

The primary dataset that was used for these experiments consisted of 715 instances of patients (30 clinical + imaging features) who underwent nuclear stress myocardial perfusion imaging and was obtained through the Cedars-Sinai Medical Center (CSMC) Artificial Intelligence in Medicine Program^6^ (Supplement Table S3). The retrospective use of clinical data in this study was approved by the Institutional Review Board at Cedars-Sinai and informed consent was obtained from all patients. Preprocessing for missing/null data was addressed through the deletion of rows, ultimately leaving 681 final instances to be used for the machine learning algorithms. Mean column imputation was also performed, with the corresponding results in the supplement. The binary label in the data set used to train the classification algorithms was whether the patient required a revascularization procedure following the nuclear imaging test, which occurred in 373 (54%) patients. To protect patient privacy, all information was deidentified.

The secondary dataset used for evaluation included 2691 patients from 1 centre in the prognostic cohort of the REFINE SPECT registry^7^. Patients were consecutively recruited at their respective centres with no previously known coronary artery disease (CAD), myocardial infarction, or previous coronary revascularization. The retrospective use of clinical data in this study was approved by the Institutional Review Board at each of the respectives centres and Cedars Sinai. Informed consent was obtained from all patients. We specifically used 18 clinical variables and 9 stress-test variables to predict major adverse cardiac events (MACE) which occurred in 522 (19%) patients (Supplement Table S4). This is a common outcome endpoint in clinical cardiology. Mean column imputation was performed for missing values in this dataset.

Both studies were performed in accordance with the ethical standards of the Declaration of Helsinki (1964) and its subsequent amendments.

### Test/validation regimen

For implementing 50/50 split validation, 100 different models (each using a unique seed) were built and the AUCs were calculated for each model. For implementing the 70/30 split validation, the method was nearly identical, except for the change in the fractions of the data allocated to the test and training sets. For tenfold stratified CV, rather than averaging the AUCs for each fold, the actual and predicted probabilistic values for each seed across all the folds were concatenated into two global arrays, from which an accurate global AUC could be calculated. The more comprehensive 10 × repeated tenfold CV was implemented similarly; however, rather than concatenating all the results of the folds of a single seed, it concatenated the results of 100 folds accumulated over 10 different seeds. Both cross-validation methods and the split validation methods involved stratification, to ensure a consistent distribution of labels in both training and test splits. The bootstrap sampling was used to generate a training set equal to the number of samples, while the validation set consisted of the samples that were not selected for the training set. The AUC was averaged from all 500 repetitions of bootstrap sampling, and the confidence intervals were computed from the concatenation of the predicted and actual values through these iterations using the Delong method^18^.

### Evaluation

For each validation experiment (defined as an algorithm paired with a validation method), the max and min AUCs were recorded (representing the maximum variation in the experiment). Statistical significance between the max and min cases was measured using the VassarStats statistical comparison (non-directional) between two independent ROC curves^8^. The ROC curve plots the false positive rate (FPR) against the true positive rate (TPR) as a function of the decision threshold (0 to 1). We define the FPR and TPR below:

$$FPR = ~\frac{{FP}}{{TN + FP}}\;\;\;~TPR = ~\frac{{TP}}{{TP + FN}}$$

The AUC of this curve provides a measure of classification performance that includes the full range of possible decision thresholds. Although we used the same pool of original data, causing overlap in training data to build the models, the nature of the seed variation prevents us from using the normal Delong-Delong comparison since the training and test sets consistently vary and cannot be paired. To address this issue, we treat these samples as independent. We additionally assessed Leave One Out (LOO) Validation, which has no stochasticity from split variation, and thus no minimum/maximum for the sake of comparison with methods involving train/test splitting.

In addition to the ROC statistical comparison, a comparison of the range (difference between max and min) of the AUCs produced between the different models provides insight on the magnitude of the difference caused by random seed variation. For both datasets the 95% confidence interval for AUC was calculated for each of the maximum and minimum cases in each repeated experiment using the Delong method for confidence interval calculation^9^. A graphical representation of the ROC curves for the maximum and minimum cases was done for the gaussian naïve bayes algorithm, to get an enhanced visual understanding of how seed variation impacted the ROC curves themselves.

For the primary dataset, we additionally performed the following further analysis and experiments to understand the phenomena observed in the main experiments. We additionally performed feature selection (10 features selected) on the primary dataset using mutual information, and the range was computed when models were trained using feature selection. We used mean column imputation as well to understand how that impacted the range results as it increased the sample size by 5%. Although the primary focus of the paper is to evaluate the instability of ROC model evaluation metrics from split variation, we additionally evaluated the variation in accuracy and F1 score. To evaluate computational feasibility, CPU execution time (single iteration) was measured using Python’s cProfile module, a tool for evaluating how Python source consumes processor resources. This time includes the full pipeline from dataset loading to performing predictions in each case and was done with a 2.7 GHz Intel “Core i5” processor.

## Results

For the stratified split-sample validation techniques (both 50/50 and 70/30) across all four algorithms and in both datasets (Cedars Sinai and REFINE SPECT Registry), a comparison between the ROC curves generated for the seeds with the maximum and minimum AUC revealed statistically significant results (p < 0.05) (Tables 1, 2). These ROC curves were not significantly different when using tenfold stratified CV, 10 × repeated stratified tenfold CV, and bootstrap validation.

**Table 1:** 95% AUC estimate/confidence intervals (CI) for maximum and minimum cases (Cedars).

Method	Bootstrap validation	50/50 strat split validation	70/30 strat split validation	tenfold strat. cross val	10 × repeated tenfold strat cross val
Logistic regression: Max	0.783 [0.778–0.783]	0.833** [0.789–0.877]	0.853** [0.801–0.904]	0.802 [0.769–0.835]	0.797 [0.787–0.808]
Logistic regression: Min	0.778 [0.772–0.778]	0.739** [0.687–0.792]	0.726** [0.657–0.794]	0.783 [0.749–0.818]	0.791 [0.781–0.803]
Gaussian Naïve Bayes: Max	0.766 [0.748–0.753]	0.815** [0.769–0.861]	0.850** [0.798–0.903]	0.771 [0.735–0.806]	0.767 [0.756–0.778]
Gaussian Naïve Bayes: Min	0.761 [0.748–0.754]	0.721** [0.667–0.775]	0.687** [0.614–0.760]	0.750 [0.713–0.787]	0.760 [0.749–0.772]
Linear discriminant analysis: Max	0.781 [0.776–0.781]	0.821* [0.777–0.866]	0.850** [0.798–0.902]	0.801 [0.768–0.833]	0.794 [0.784–0.805]
Linear discriminant analysis: Min	0.776 [0.771–0.776]	0.734* [0.682–0.786]	0.698** [0.628–0.769]	0.781 [0.747–0.815]	0.789 [0.778–0.799]
Random forest: Max	0.798 [0.793–0.798]	0.841* [0.799–0.883]	0.854* [0.802–0.906]	0.810 [0.777–0.843]	0.802 [0.792–0.813]
Random forest: Min	0.794 [0.788–0.793]	0.759* [0.708–0.810]	0.753* [0.686–0.820]	0.789 [0.755–0.824]	0.797 [0.786–0.809]

95% CI calculated using Delong method with concatenated predictions. For bootstrap validation, AUC computed as mean from the AUC of the 500 iterations, sometimes falling out of range of 95% CI. CI calculated using Delong method in brackets.

*Signifies measured statistical significance p-value < 0.05 in independent ROC comparison to max–min counterpart for the same algorithm and validation technique.

**Indicates p-value < 0.01 in independent ROC comparison. *ROC* receiver operating characteristic curve, *AUC* area under the ROC curve.

**Table 2:** 95% AUC estimate/confidence intervals (CI) for maximum and minimum cases (REFINE SPECT).

Method	Bootstrap validation	50/50 strat split validation	70/30 strat split validation	tenfold strat. cross val	10 × repeated tenfold strat cross val
Logistic regression: Max	0.792 [0.787–0.790]	0.816* [0.789–0.843]	0.834* [0.802–0.866]	0.797 [0.772–0.815]	0.795 [0.788–0.802]
Logistic regression: Min	0.788 [0.787–0.790]	0.767* [0.716–0.797]	0.756* [0.716–0.797]	0.787 [0.768–0.810]	0.792 [0.786–0.799]
Gaussian Naïve Bayes: Max	0.747 [0.694–0.698]	0.777** [0.746–0.808]	0.790** [0.752–0.828]	0.739 [0.716–0.762]	0.729 [0.722–0.736]
Gaussian Naïve Bayes: Min	0.741 [0.694–0.698]	0.708** [0.655–0.750]	0.703** [0.655–0.750]	0.705 [0.682–0.729]	0.712 [0.710–0.725]
Linear discriminant analysis: Max	0.788 [0.783–0.786]	0.817* [0.790–0.845]	0.827* [0.794–860]	0.798 [0.769–0.811]	0.795 [0.789–0.802]
Linear discriminant analysis: Min	0.785 [0.783–0.786]	0.756* [0.713–0.795]	0.754* [0.713–0.795]	0.790 [0.770–0.812]	0.792 [0.786–0.799]
Random forest: Max	0.771 [0.766–0.770]	0.801* [0.771–0.830]	0.817** [0.783–0.851]	0.784 [0.765–0.799]	0.777 [0.770–0.784]
Random forest: Min	0.768 [0.766–0.770]	0.741* [0.684–0.775]	0.730** [0.684–0.775]	0.766 [0.747–0.783]	0.772 [0.765–0.779]

95% CI calculated using Delong method with concatenated predictions. For bootstrap validation, AUC computed as mean from the AUC of the 500 iterations, sometimes falling out of range of 95% CI. CI calculated using Delong method in brackets.

*Signifies measured statistical significance p-value < 0.05 in independent ROC comparison to max–min counterpart for the same algorithm and validation technique.

**Indicates p-value < 0.01 in independent ROC comparison. *ROC* receiver operating characteristic curve, *AUC* area under the ROC curve.

**Figure 1:**
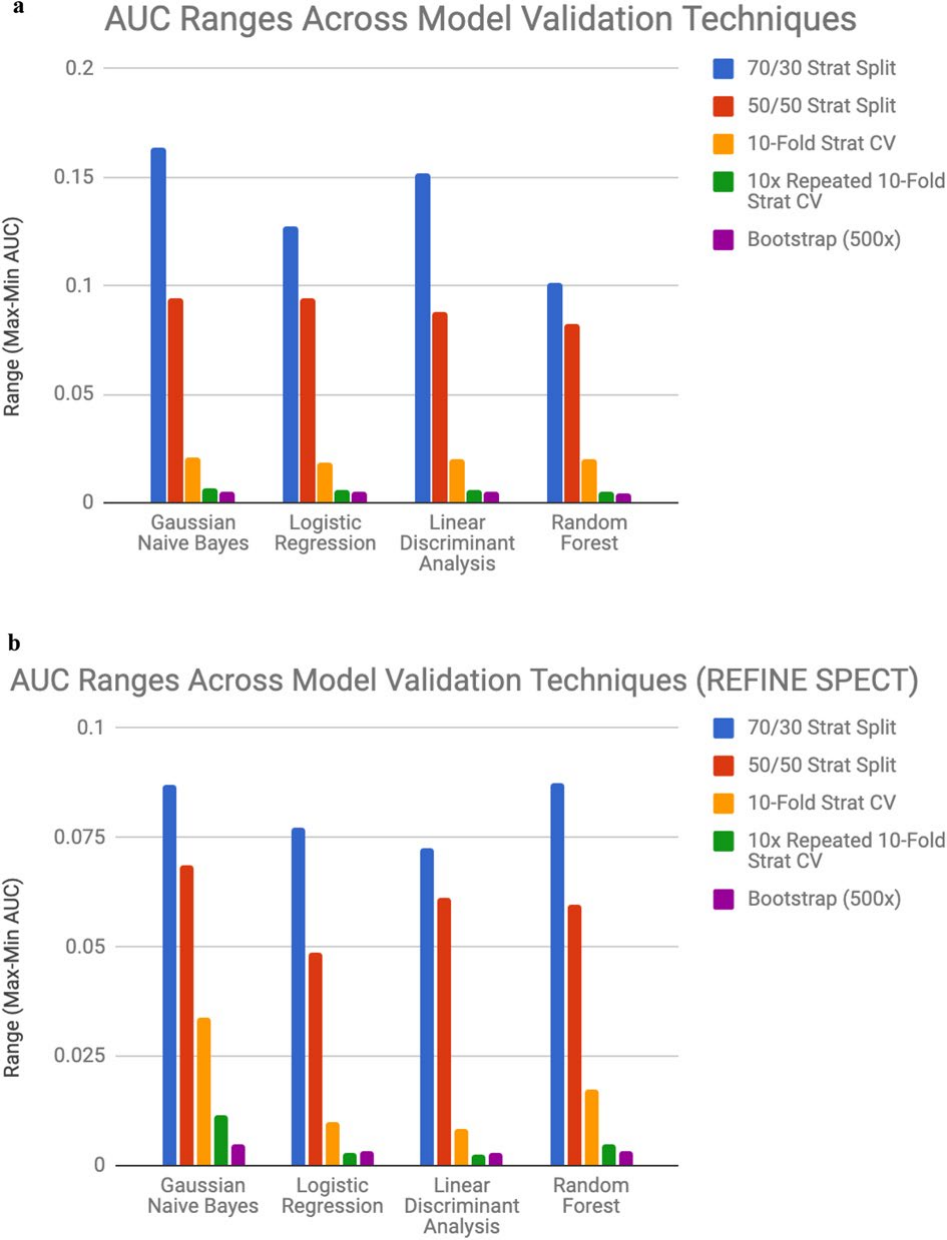
The maximum and minimum AUCs were calculated (over the 100 iterations) for each algorithm corresponding to the various model validation techniques. Their difference (range) plotted on the y-axis represents the maximum variation observed from the iterations attributed to seed alterations for the model. *AUC* area under the ROC curve. *ROC* receiver operating characteristic curve. *CV* cross-validation. *Strat* stratified. **(a)** Primary Cedars Sinai dataset, **(b)** Secondary SPECT REFINE dataset.

Observing the range (the difference between max and min of all the 100 AUCs in each experiment) adds to the story, demonstrating sharp declines when using tenfold stratified cross-validation compared to the split validation techniques, and even sharper declines when using the comprehensive 10 × repeated tenfold stratified CV and bootstrap validation in both datasets (Fig. 1). In the primary Cedars Sinai dataset, the generated 95% confidence interval for the AUC estimates for the maximum and minimum cases further demonstrate this trend, as the ranges tend to get tighter in the tenfold CV, 10 × repeated tenfold stratified CV, and bootstrap validation in comparison to the split validation approaches (Tables 1, 2). The increased precision (stability of results over repeated simulations with seed variation) of the 10 × repeated tenfold validation and bootstrap validation is observed when comparing the range in AUCs across seeds using the different model validation techniques, producing more stable results in comparison to even the standard tenfold stratified cross-validation (Fig. 1).

The leave-one-out validation (which did not have any random split variation) led to similar performance estimates as the tenfold stratified CV, repeated tenfold stratified CV, and bootstrap validation methods in both evaluated datasets (Supplement Tables S1, S2). Both using mutual information feature selection to reduce the feature space to 10 variables and using mean column imputation led to a similar range in AUC estimates in the Cedars dataset with the variation remaining statistically significant in all the split validation scenarios (Supplement Table S5, Supplement Figs. S3, S4). Both the accuracy and F1 scores had notable variation from split variation in the primary, although the primary emphasis of this paper was the AUC estimates, the standard in cardiovascular imaging (Supplement Figs. S1, S2).

**Figure 2:**
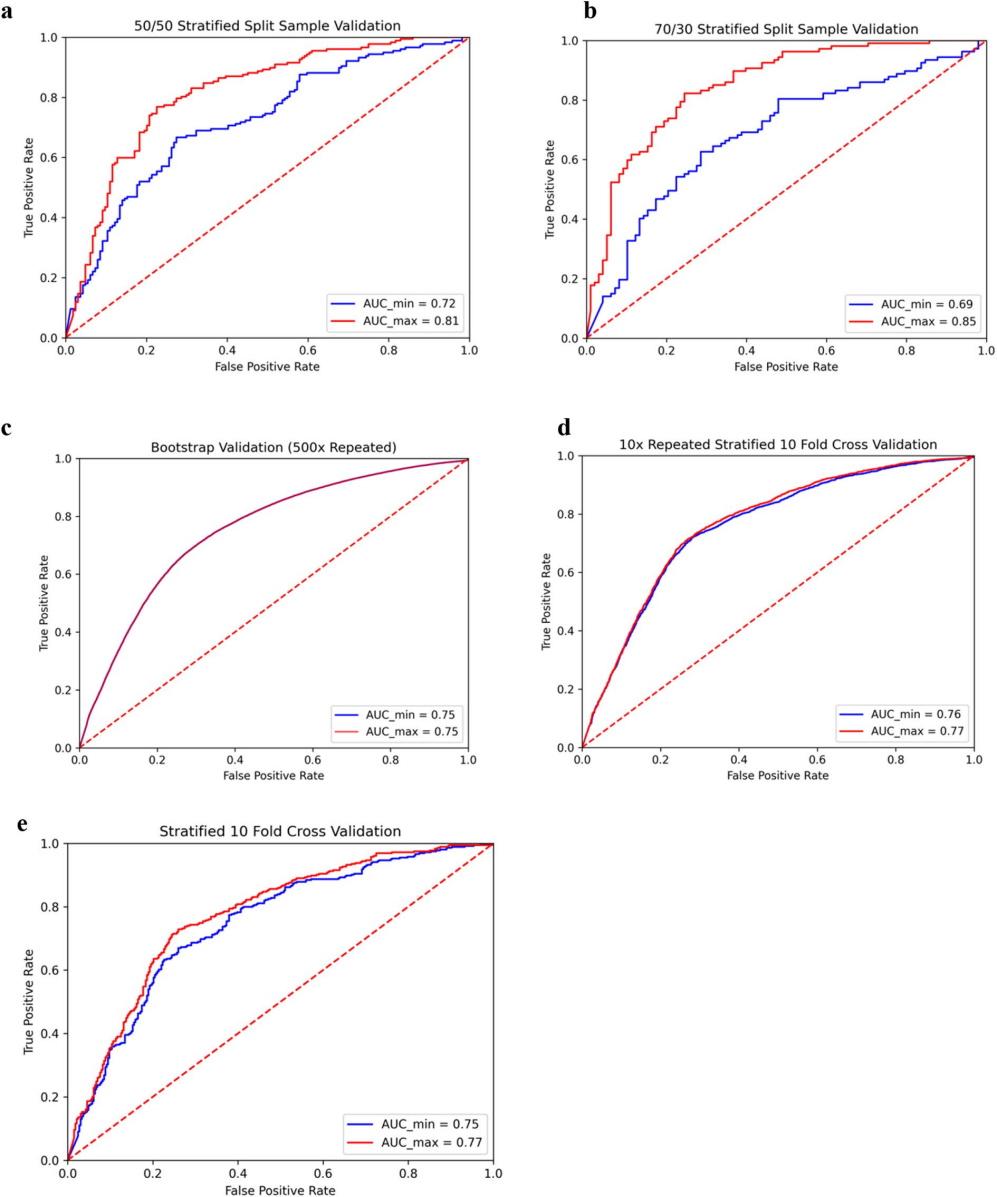
For experiments run using the Gaussian Naïve Bayes algorithm, two ROCs were generated for each validation technique (for the max and min AUCs) in order to visually observe the impact that seed changes could have on the ROC curve itself. **(a)** 50/50 Stratified Split Sample Validation. **(b)** 70/30 Stratified Split Sample Validation. *ROC* receiver operating characteristic curve. **(c)** Bootstrap Validation (500 × Repeated) 10-Fold Stratified CV. **(d)** 10 × Repeated 10-Fold Stratified CV. **(e)** 10-Fold Stratified CV. *ROC* receiver operating characteristic curve. *AUC* area under the ROC curve. *ROC* receiver operating characteristic curve.

The split validation techniques required the shortest execution time, whereas the tenfold stratified CV, 10 × repeated tenfold stratified CV, and bootstrap validation (500 ×) significantly increased in that respective order (Table 3). For all these methods however the execution time was within reason, especially given the sample size of datasets typically used for applied machine learning in nuclear cardiology. Computational constraints should not be a limiting factor for performing more rigorous validation methods for obtaining more robust performance estimates of machine learning models for datasets of this size. For experiments done using the Gaussian Naïve Bayes algorithm specifically, the ROC curves for max and min cases were produced for each of the validation techniques aligned with the numerical results, with a large difference between curves produced for the split validation techniques and nearly identical curves for the more rigorous 10 × repeated tenfold stratified CV and bootstrap validation methods (Fig. 2, Supplement Fig. S5).

**Table 3:** CPU execution time for each validation mechanism (random forest/cedars Sinai dataset).

Validation mechanism	CPU execution time (s)
50/50 stratified split validation	1.651
70/30 stratified split validation	1.624
tenfold stratified CV	3.775
10 × repeated tenfold stratified CV	25.955
Bootstrap validation (500x)	124.600
Leave one out validation	114.492

*CPU* central processing unit, *CV* cross-validation.

## Discussion

This study aimed to analyze how the selection of model validation technique impacted the stability of ROC-related performance estimates. Our experiments demonstrate that random seed selection (case selection) can alter performance estimates in a statistically significant fashion when a machine learning model is being validated using split-sample validation techniques (both 70/30 and 50/50). This was observed in both the Cedars Sinai dataset for prediction of revascularization (n = 715) and the larger prognostic dataset (n = 2691) in predicting the major adverse cardiovascular events. While the range in the AUC was smaller in the larger secondary dataset, the induced variation across all four algorithms when using 50/50 stratified split validation and 70/30 stratified split validation was nevertheless statistically significant (Fig. 1). While larger variation is expected when using split validation techniques compared to the more rigorous validation techniques, this result demonstrates how statistically significant variation can be induced from the split alone. Although more computationally expensive, tenfold stratified CV, especially 10 × repeated tenfold stratified CV along with bootstrap validation, provide higher precision and stability to performance estimates as evidenced through narrower ranges in performance estimate extrema across repeated simulations and visualization of ROC curves corresponding to these extrema.

In the field of cardiology, split-validation has been a default method when evaluating machine learning models, despite newer more rigorous techniques such as CV being introduced as having more validation power^10^. In the industry, hold-out data sets often embody this same concept of split validation, which is vulnerable to the same demonstrated inconsistencies presented, especially with a smaller sample size. Although bootstrap validation is starting to become less common in the field, it is still used in the field and serves to illuminate how sampling with replacement in the selection of training and test sets alters the outcomes of the simulations in comparison to other methods^11^. Although bootstrap tends to have low variability due to the large number of estimates averaged in each iteration as demonstrated in our study, it has been shown in other simulation studies to have a noticeable bias in smaller sample sizes^12^. The results of this study highlight the instability and vulnerability of performance estimates to issues of statistical significance comparisons when applying split-validation on machine learning models built on two real-world datasets of varying sizes in cardiology. There are several examples of papers published on applied machine learning in medicine with similar sample sizes to the one used in this study. When researchers applied machine learning to predict presence of obstructive coronary artery disease (CAD) in patients who underwent diagnostic coronary angiography, from the Catheter Sampled Blood Archive in Cardiovascular Diseases [CASABLANCA] Study (n = 927, 64% CAD rate), a split validation set was used to calculate the AUC and even compare the performance of predictive models created solely with clinical variables and those with biomarkers and clinical variables^15^. In a study applying machine learning to predict survival in candidates for HeartMate II (HMII), left ventricular assist device (LVAD) support (n = 1122, 13% mortality rate), the patients were randomly divided into derivation and validation cohorts^16^. The median sample size in model development studies being only 445 subjects demonstrates the logistical challenges in accumulating very large sample sizes for model development studies with human subjects^17^. The lack of larger sample sizes makes the validation technique even more critical in these studies in terms of deriving insights and making comparisons using machine learning models. Although some argument can be made for inducing more stability on the algorithm side through meta-algorithms such as ensemble learners^13^, the experimental results showing instability in performance estimates also holds for experiments done using the random forest algorithm (with bagging).

This study has several limitations. In our experiments, we used a relatively small data set (681 instances) and a mid-sized dataset (2691 instances); however, in the medical field, it is often not possible to have large sample sizes for data sets, and this limited sample size represents a common, real life challenge for researchers when applying machine learning to clinical data sets^14^. The differences between split-sample and CV would be less pronounced in larger populations; we did not evaluate this effect in larger data samples in this study. Another validation mechanism that was not examined in this paper that has been discussed in the 3-step process of training-validation-test, in which the data is broken into three sets, each for training, validation, and testing. The model is tuned using the validation set (learning via training set), with the test set being used to assess the performance (generalization) to confirm the actual predictive power of the model. Analyzing the impact of seed variation on this approach is intriguing for future study. While different choices of k (also commonly 5) could be used in the k-fold stratified CV and repeated k-fold stratified CV to reduce validation time, we use 10 due to the small sample size of the datasets used in these studies, which allow higher k values to be used without compromising reasonable validation times.

Overall, the results of this study exhibit the instability of ROC-related performance estimates on two real-world clinical datasets and favor alternative stratified CV mechanisms and bootstrap validation mechanisms for reliable performance evaluation of machine learning models. When attempting to assess the impact of a particular independent variable, it is demonstrated on this dataset that seed can be another independent variable capable of significantly impacting the results, creating an unwanted confounding effect. Split-sample validation techniques are traditionally used in model evaluation in medicine, so the implications of this study can have a real impact on moving towards stronger practice of validation techniques for applied machine learning and statistical modeling in the medical field.

## Supplementary Information

Supplementary Information.

## Data Availability

The supporting data can be made available upon written request to the corresponding author.
