# Supplementary material for: Impact of train/test sample regimen on performance estimate stability of machine learning in cardiovascular imaging
Source: Sci Rep. 2021 Jul 14;11:14490. doi: 10.1038/s41598-021-93651-5 (PMC8280147; doi:10.1038/s41598-021-93651-5)
Supplement: Supplementary file 1 — Supplementary Information. [file 41598_2021_93651_MOESM1_ESM.docx]

**Supplement Table 1. 95% Leave One Out Validation Model Evaluation Metrics (Cedars-Sinai)**

|  | **AUC** | **Accuracy** | **F1-Score** |
| --- | --- | --- | --- |
| **Logistic Regression** | 0.797  [0.7573-0.825] | 0.730 | 0.734 |
| **Gaussian Naive Bayes** | 0.762  [0.7255-0.798] | 0.665 | 0.617 |
| **Linear Discriminant Analysis** | 0.792  [0.7587-0.8258] | 0.724 | 0.729 |
| **Random Forest** | 0.804  [0.7705-0.837] | 0.736 | 0.746 |

**95% CI for AUC calculated using Delong method in brackets**

**Supplement Table 2. 95% Leave One Out Validation Model Evaluation Metrics (REFINE SPECT Registry)**

|  | **AUC** | **Accuracy** | **F1-Score** |
| --- | --- | --- | --- |
| **Logistic Regression** | 0.795  [0.7741-0.8158] | 0.822 | 0.368 |
| **Gaussian Naive Bayes** | 0.745  [0.7219-0.7678] | 0.786 | 0.254 |
| **Linear Discriminant Analysis** | 0.794  [0.7731-0.8149] | 0.825 | 0.393 |
| **Random Forest** | 0.772  [0.7502-0.7947] | 0.821 | 0.312 |

**95% CI for AUC calculated using Delong method in brackets**

**Supplement Table 3: Dataset Features (Cedars-Sinai)**

| Sex |
| --- |
| Age |
| Diabetes |
| Cholesterol |
| Smoking |
| Family History |
| Hypertension |
| Claudication |
| Height |
| Weight |
| BMI |
| Symptom |
| Clinical Response |
| ECG Response |
| ST Segment Depression (ECG) |
| Heart Rate Supine |
| Blood Pressure Supine |
| Heart Rate Peak |
| Blood Pressure Peak |
| Exercise Duration |
| Metabolic Equivalent of Task |
| Transischemic Dilation Ratio |
| Pretest Probability of Coronary Disease |
| Post ECG Probability of Coronary Disease |
| Quantitative Total Perfusion Deficit |
| Q_STR_TPD_P+ Quantitative Combined Supine Prone Stress Total Perfusion Deficit |
| Q_STR_TPD Quantitative Stress Total Perfusion Deficit |
| Q_RST_TPD Quantitative Rest Total Perfusion Deficit |
| P+_STR_TPD Combined Supine Prone Stress Total Perfusion Deficit |
| P+_RST_TPD Combined Supine Prone Rest Total Perfusion Deficit |

**Supplement Table 4: Dataset Features (REFINE SPECT Registry)**

| **Clinical Variables** |
| --- |
| BMI |
| Gender |
| Rest SBP (mmHg) |
| Indication for Test |
| Age |
| Symptoms (1, 2, 3, 4) |
| Conduction Disease (0, 1, 2, 3, 4, 5) |
| Location (inpatient, outpatient, ed) |
| Under Drug Influence (0, 1, 2, 3, 4, 5) |
| Left Ventricular Hypertrophy (0, 1) |
| Rest Heart Rate (beats/minute) |
| Dyslipidemia (0,1) |
| Peripheral Vascular Disease (0, 1) |
| Family History (0, 1) |
| Diabetes Mellitus (0, 1) |
| Abnormal Rest ECG (0, 1) |
| Hypertension (0, 1) |
| Current Smoker (0, 1) |
| **Stress Test Variables** |
| ST Deviation (mm) |
| ECG Response to Stress (1, 2, 3, 4, 5) |
| Stress Peak Heart Rate (beats/minute) |
| Test Termination Reason (1-11) |
| Clinical Response to Stress (1, 2, 3, 4, 5) |
| Stress Test Type (1, 2, 3, 4, 5) |
| Pharmacologic Stress Agent (1, 2, 3, 4, na) |
| Stress Peak SBP (mmHg) |
| Exercise Duration (Minutes) |

**Supplement Table 5: Top 10 Features Ranked (Cedars Sinai) using Mutual Information with Target Variable**

| 1. Quantitative Stress Total Perfusion Deficit |
| --- |
| 1. Quantitative Combined Supine Prone Stress Total Perfusion Deficit |
| 1. Combined Supine Prone Stress Total Perfusion Deficit |
| 1. ECG Response |
| 1. Sex |
| 1. Diabetes |
| 1. Symptom |
| 1. Hypertension |
| 1. Claudication |
| 1. Quantitative Rest Total Perfusion Deficit |


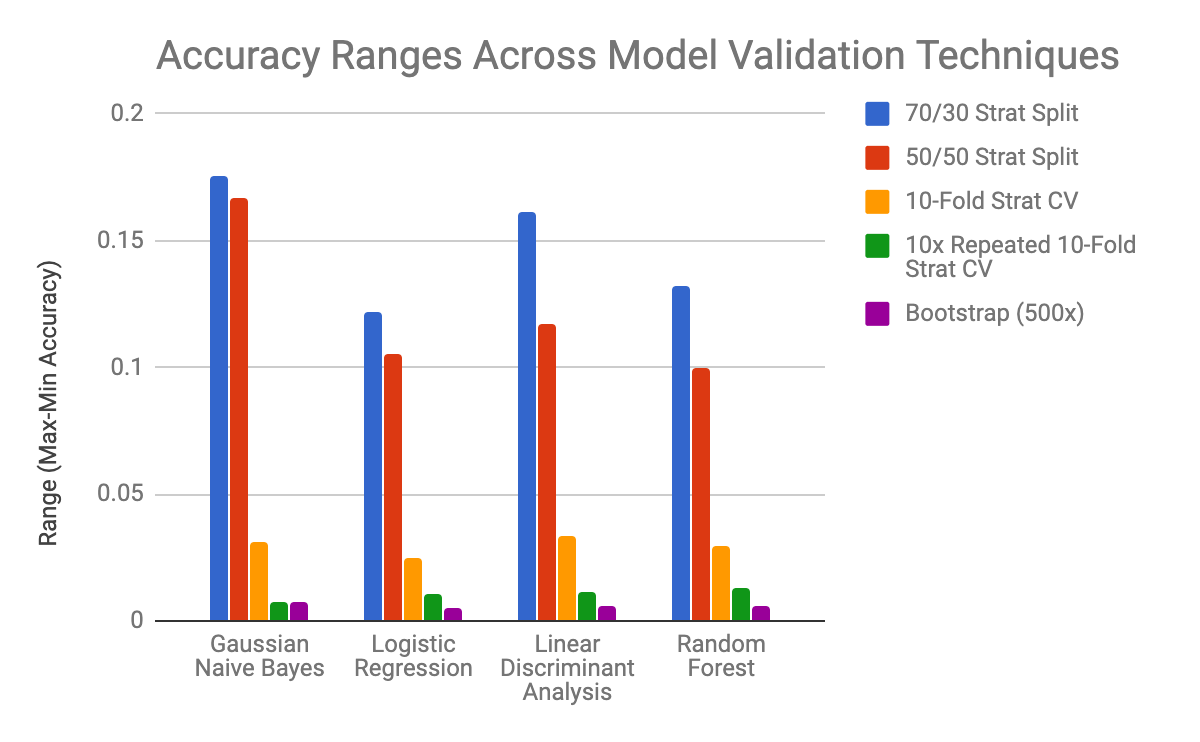


**Supplement Figure 1.** The maximum and minimum accuracy scores were calculated in the Cedars dataset (over the 100 iterations) for each algorithm corresponding to the various model validation techniques. Their difference (range) plotted on the y-axis represents the maximum variation observed from the iterations attributed to seed alterations for the model.


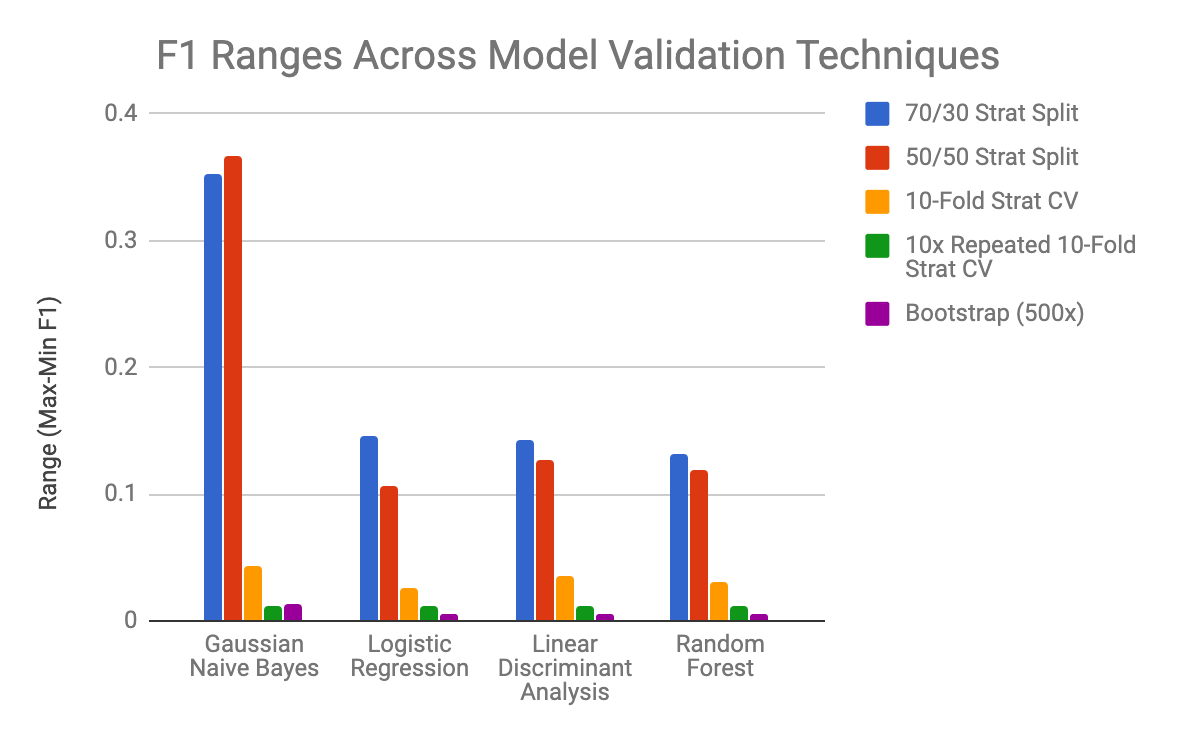


**Supplement** **Figure 2.** The maximum and minimum F1 scores were calculated in the Cedars dataset (over the 100 iterations) for each algorithm corresponding to the various model validation techniques. Their difference (range) plotted on the y-axis represents the maximum variation observed from the iterations attributed to seed alterations for the model.


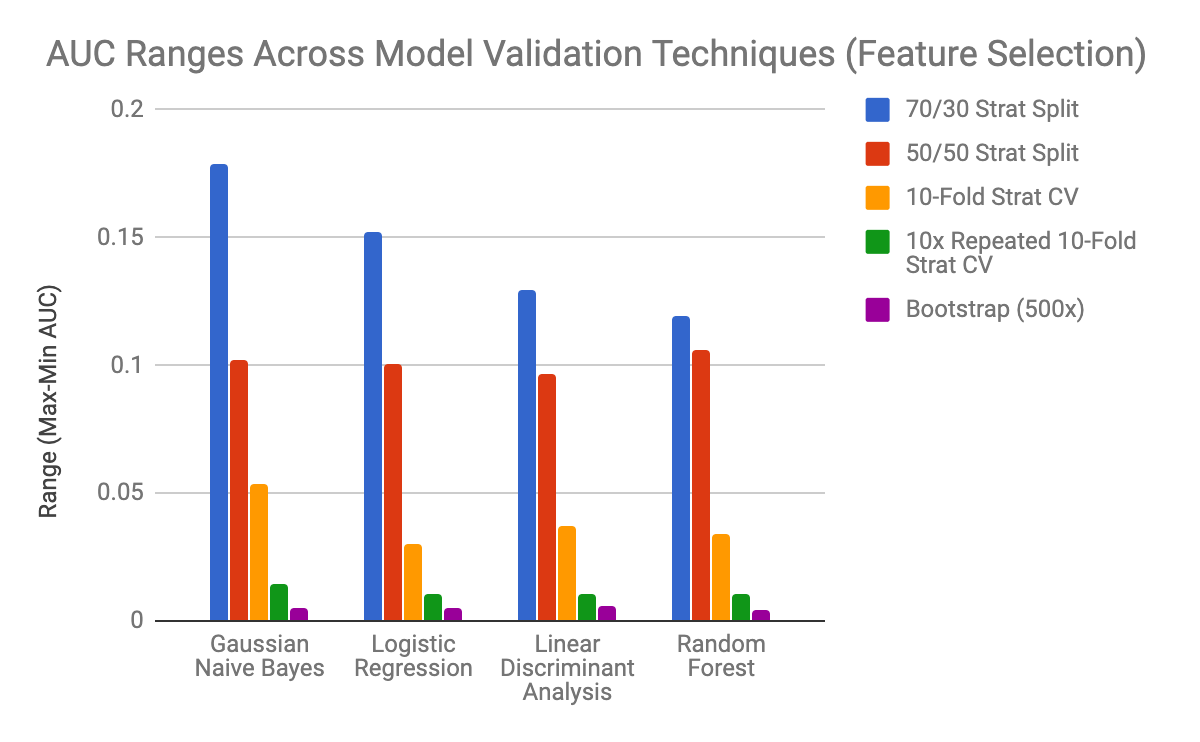


**Supplement** **Figure 3.** The maximum and minimum AUC scores were calculated in the Cedars dataset (over the 100 iterations) for each algorithm corresponding to the various model validation techniques using the top 10 features selected through mutual information feature selection (highest mutual information with the target variable). Their difference (range) plotted on the y-axis represents the maximum variation observed from the iterations attributed to seed alterations for the model.


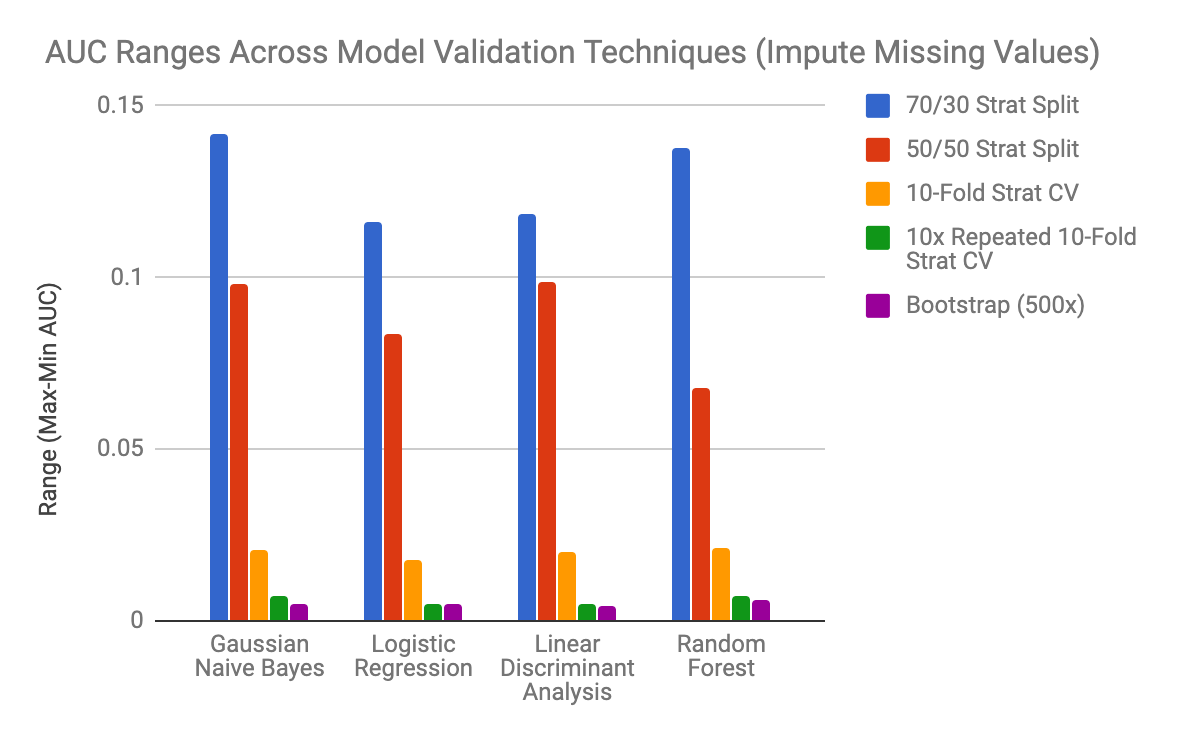


**Supplement Figure 4.** The maximum and minimum AUC scores were calculated in the Cedars dataset (over the 100 iterations) for each algorithm corresponding to the various model validation techniques with column mean imputation performed to handle missing values. Their difference (range) plotted on the y-axis represents the maximum variation observed from the iterations attributed to seed alterations for the model.

**a** **b**


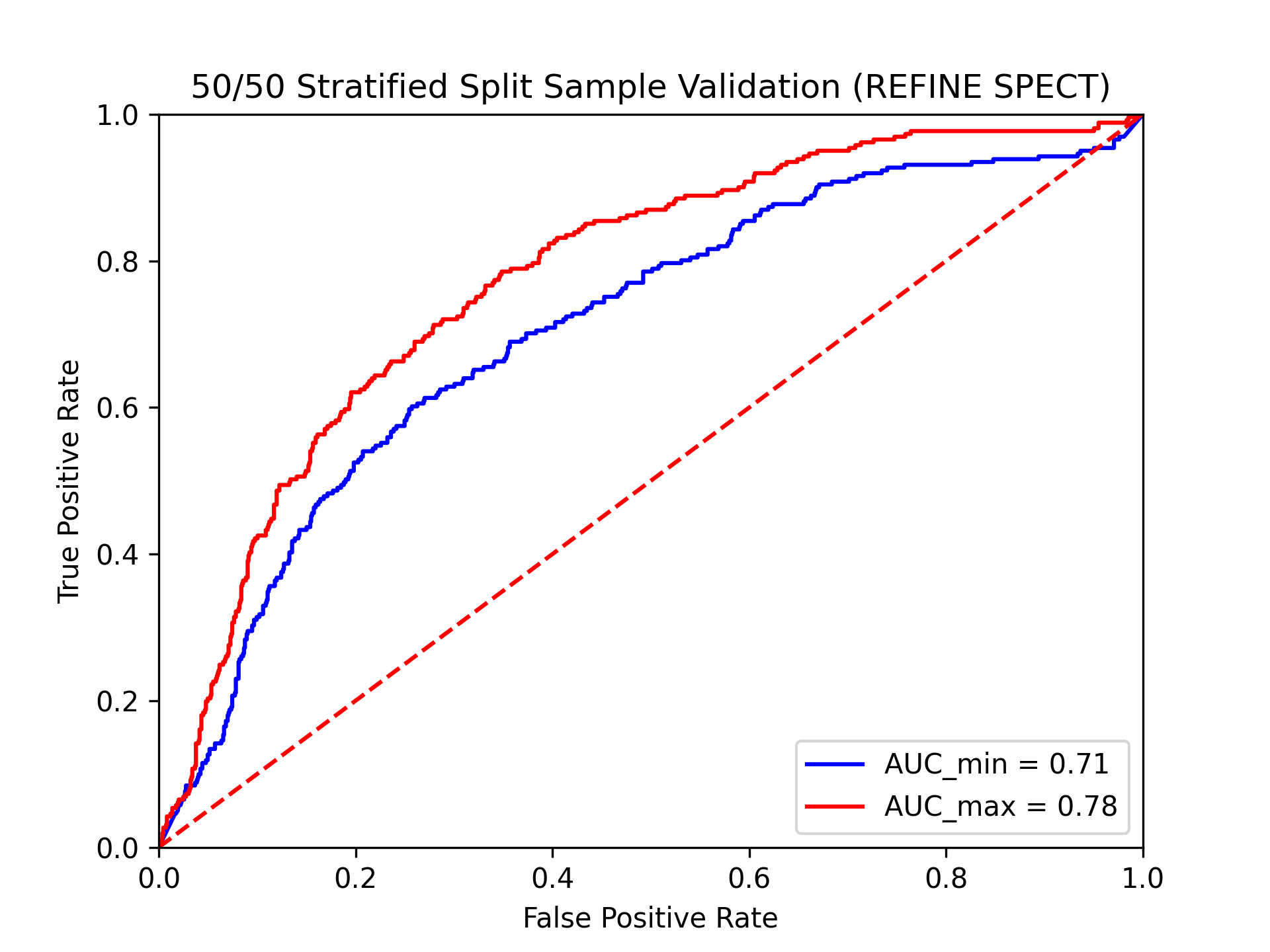

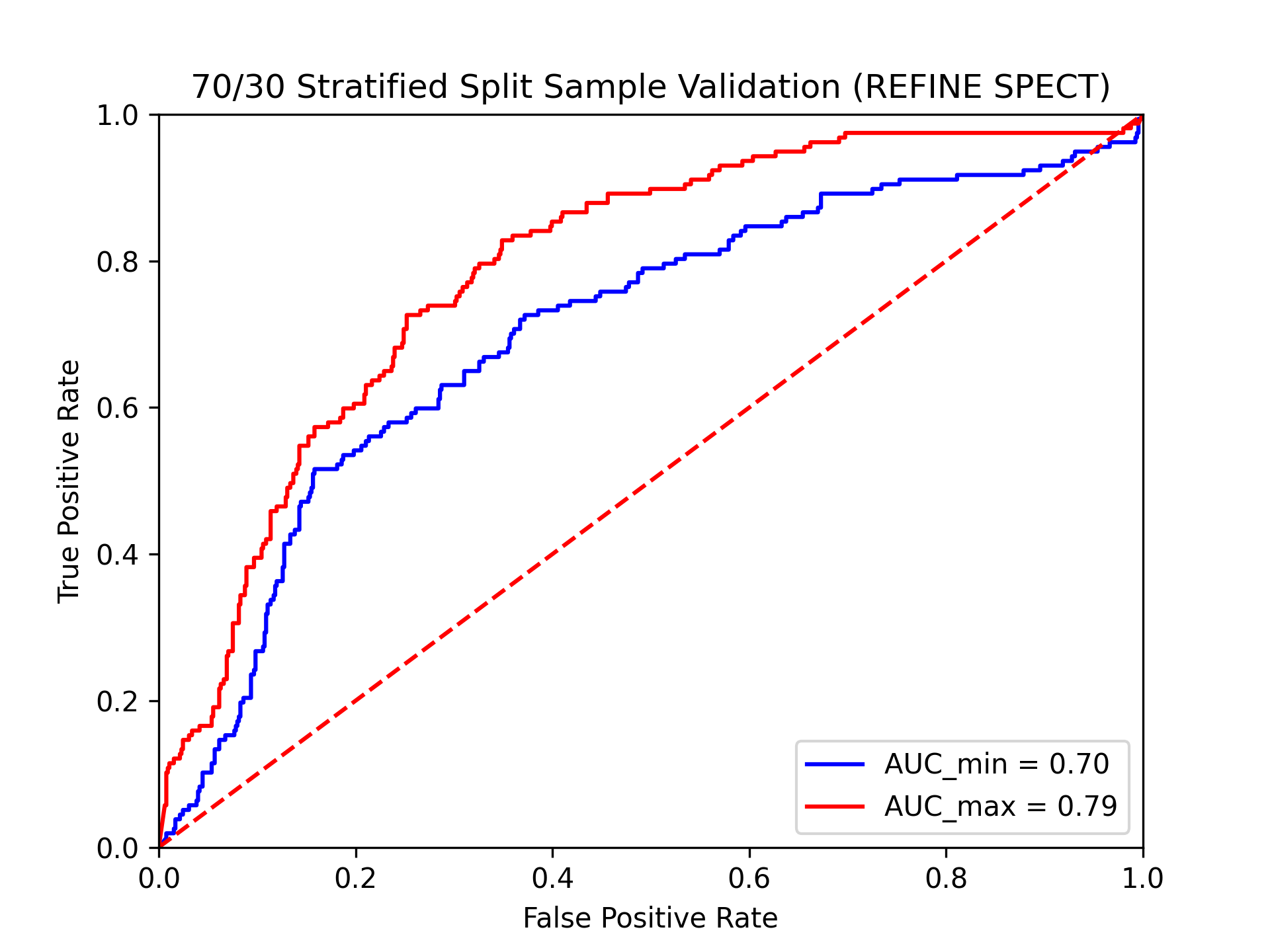


**c d**


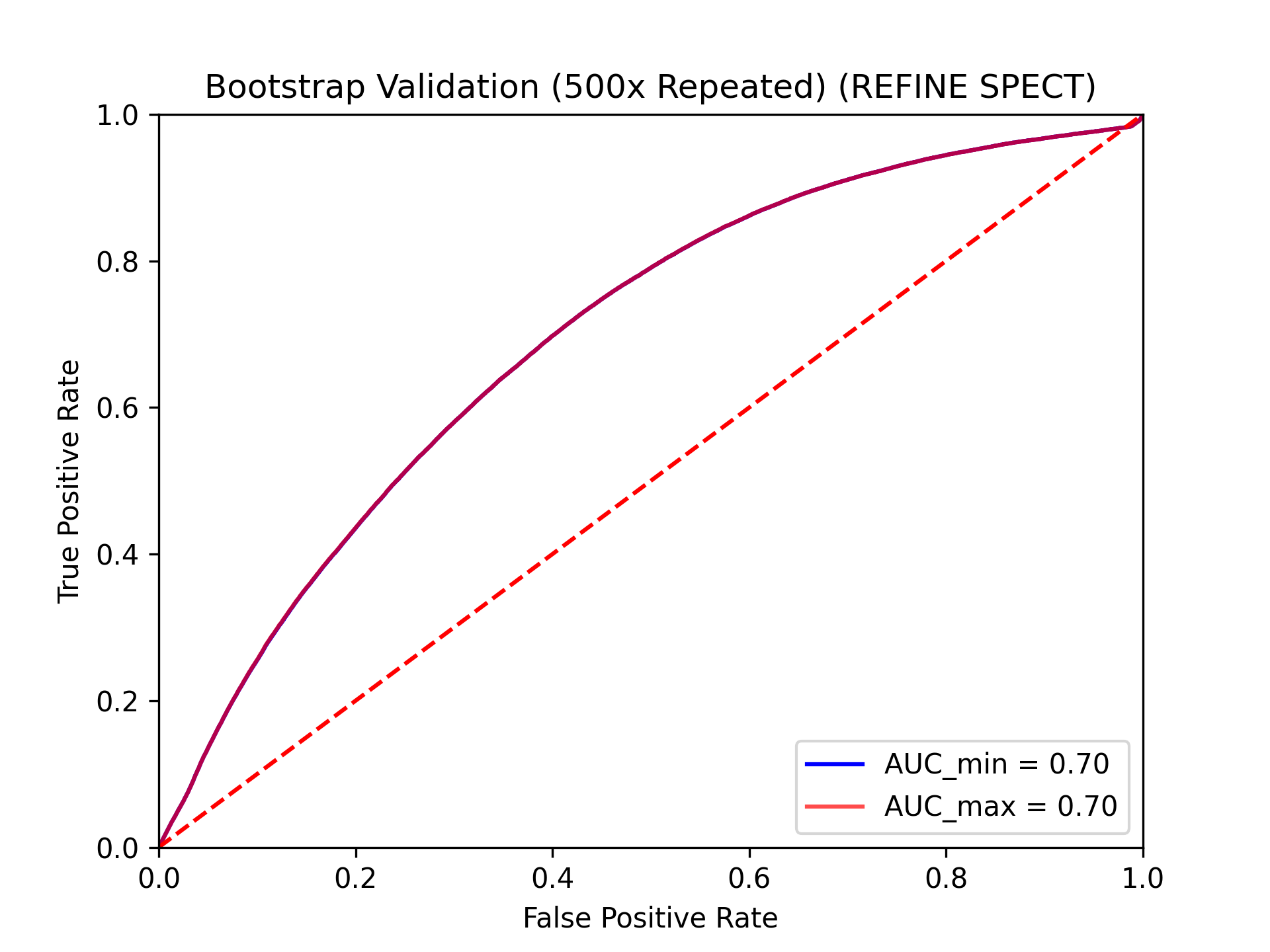

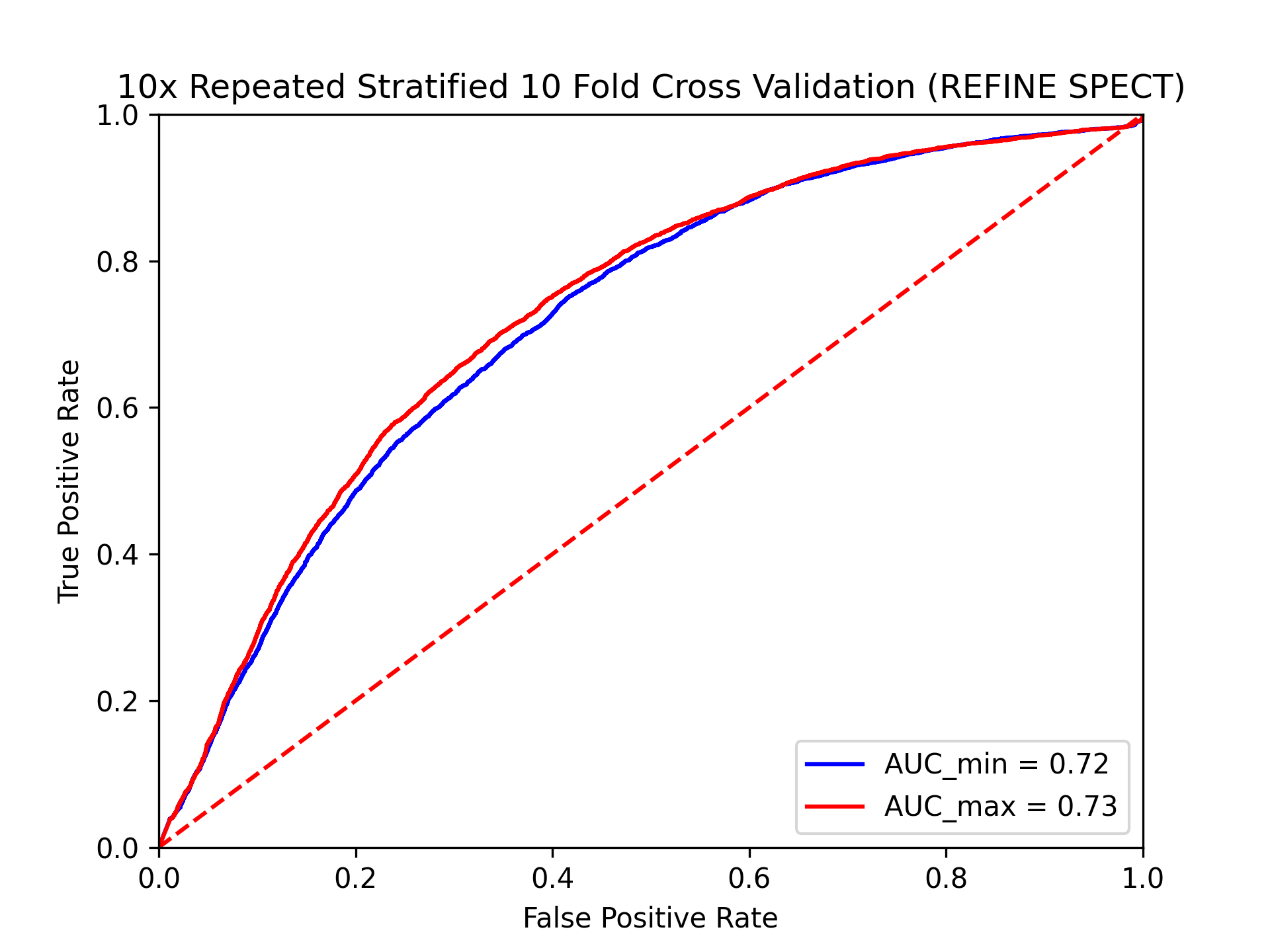


**e**


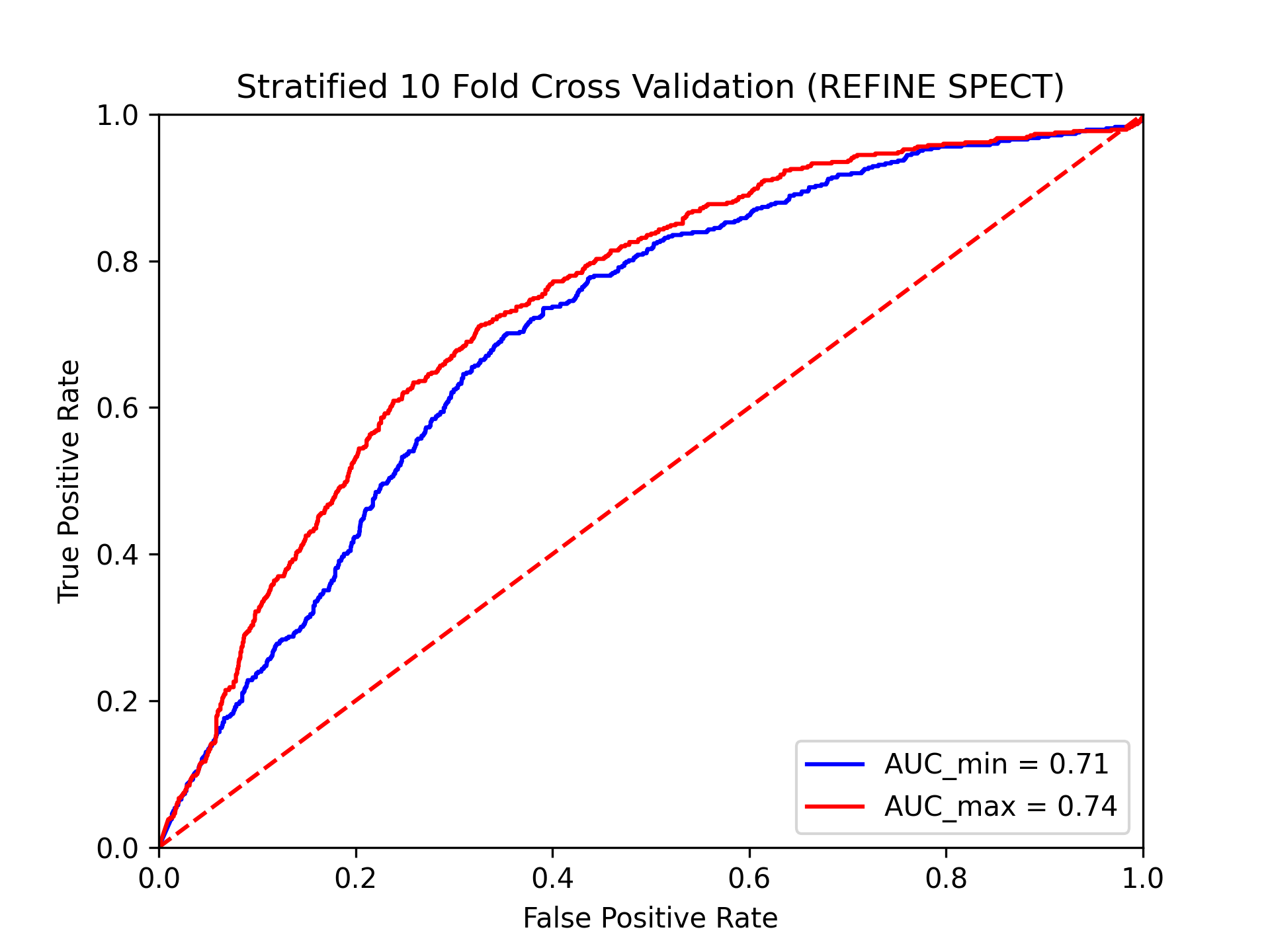


**Supplement Figure 5.** For experiments run using the Gaussian Naïve Bayes algorithm (REFINE SPECT dataset), two ROCs were generated for each validation technique (for the max and min AUCs) in order to visually observe the impact that seed changes could have on the ROC curve itself. **(a)** 50/50 Stratified Split Sample Validation. **(b)** 70/30 Stratified Split Sample Validation. ROC: receiver operating characteristic curve. **(c)** Bootstrap Validation (500x Repeated) 10-Fold Stratified CV. **(d)** 10x Repeated 10-Fold Stratified CV. **(e)** 10-Fold Stratified CV. ROC: receiver operating characteristic curve. AUC: area under the ROC curve. ROC: receiver operating characteristic curve.
